# Homogentisic Acid and Gentisic Acid Biosynthesized Pyomelanin Mimics: Structural Characterization and Antioxidant Activity

**DOI:** 10.3390/ijms22041739

**Published:** 2021-02-09

**Authors:** Maher Al Khatib, Jessica Costa, Daniele Spinelli, Eliana Capecchi, Raffaele Saladino, Maria Camilla Baratto, Rebecca Pogni

**Affiliations:** 1 Department of Biotechnology, Chemistry and Pharmacy, University of Siena, Via A. Moro 2, 53100 Siena, Italy; maher.al@unisi.it (M.A.K.); jessica.costa2@unisi.it (J.C.); mariacamilla.baratto@unisi.it (M.C.B.); 2 Next Technology Tecnotessile, Via del Gelso 13, 59100 Prato, Italy; chemtech@tecnotex.it; 3 Department of Ecology and Biology, University of Tuscia, 01100 Viterbo, Italy; e.capecchi@unitus.it (E.C.); saladino@unitus.it (R.S.)

**Keywords:** homogentisic acid, gentisic acid, laccase, pyomelanin mimics, radical species, EPR, antioxidant activity

## Abstract

Pyomelanin mimics from homogentisic acid (HGA) and gentisic acid (GA) were biosynthesized by the oxidative enzyme *T. versicolor* laccase at physiological pH to obtain water soluble melanins. The pigments show brown-black color, broad band visible light absorption, a persistent paramagnetism and high antioxidant activity. The EPR approach shows that at least two different radical species are present in both cases, contributing to the paramagnetism of the samples. This achievement can also shed light on the composition of the ochronotic pigment in the Alkaptonuria disease. On the other hand, these soluble pyomelanin mimics, sharing physico-chemical properties with eumelanin, can represent a suitable alternative to replace the insoluble melanin pigment in biotechnological applications.

## 1. Introduction

Melanins are pigments naturally occurring in all species of the biological kingdoms. In humans, melanins are generated through the enzymatic oxidation and polymerization of tyrosine and in lower organisms by the autooxidation of phenolic compounds [1,2,3]. There are different categories of melanins—including eumelanins, pheomelanins, and allomelanins. The most important form of melanin in humans is the insoluble brown-black pigment of eumelanin which represents the pigment responsible of the colour and photoprotection of the skin [2]. On the other hand, allomelanins are a heterogeneous group of melanins including pyomelanin. Pyomelanin is generated by the catabolism of tyrosine or phenylalanine through the activity of the enzyme 4-hydroxyphenylpyruvic acid dioxygenase (4-HPPD) and homogentisic acid oxidase (HGA-oxidase). When the HGA-oxidase is absent, there is an overproduction of HGA with the subsequent autoxidation and self-polymerization originating the pyomelanin pigment [3]. In humans, the genetic disorder alkaptonuria is related to a deficiency of the enzyme homogentisate 1,2 dioxygenase. This leads to the accumulation of HGA monomer which, then, for spontaneous autoxidation to 1,4-benzoquinone-2-acetic acid, polymerizes into the characteristic ochronotic pigment with not yet clearly identified structure [4,5]. All melanins exhibit interesting physico-chemical properties associated with their broad band visible light absorption, a persistent paramagnetism related to their free radical properties, antioxidant activity, and conduction properties [6]. In recent years, melanins have been proposed for different biotechnological applications in optoelectronics, biomaterials functionalization, and biomedical applications [7,8,9,10]. However, its insolubility hampers a homogeneous tissue distribution. Alternatively to the insoluble melanin pigment, the research of new soluble pigments showing similar physico-chemical properties is on the way [11]. Recently, a highly soluble HGA derived pigment has been proposed for photoacoustic imaging of macrophages due to its strong absorbance that extends into near infrared (NIR) ensuring high contrast in tissues [12]. HGA derived pigments are obtained by autooxidation or via enzymatic synthesis followed by non-enzymatic polymerization. Here, we report the enzymatic synthesis of pyomelanin mimics with their spectroscopic characterization and antioxidant activity determination. Laccases (benzenediol: oxygen oxidoreductase. EC 1.10.3.2) are multicopper oxidases able to oxidize a wide variety of substrates with the concomitant reduction of oxygen to water [13]. *Trametes versicolor* laccase has been used here for the synthesis of the two pyomelanin mimics. HGA and GA are easily oxidized by the enzyme through the Marcus “outer sphere” mechanism where the difference in redox potential is the driving force for substrate oxidation [14,15,16]. The radical species then undergo an uncatalyzed coupling reaction to form polymeric structures. Biopolymers obtained by phenolic compounds are usually characterized by the presence of EPR detectable radical species. This persistent paramagnetism is attributed to the so-called comproportionation equilibria where fully reduced, fully oxidized and semi-reduced (semi-oxidized) species are present in the polymeric structure [17,18]. The continuous wave (CW) EPR technique operating at X-band is commonly used to characterize the melanin and melanin-like samples [17,19,20]. However at this frequency (ν~9 GHz), the EPR spectrum is composed by only one unresolved EPR line. In this work, different spectroscopic techniques, UV–vis, FT-IR, DLS, NMR, and CW- pulse EPR approach, have been used to characterize the pyomelanin mimics. At least two different radical species have been detected and characterized, a carbon- and an oxygen-centred radical species. Furthermore, the longitudinal relaxation times are determined and compared with that of eumelanin, through the use of the Q-band pulse EPR measurements. The two pigments are water soluble and show a high antioxidant activity.

## 2. Results

HGA and GA pyomelanin mimics (HGAm and GAm respectively) were enzymatically synthesized by the use of the *T. versicolor* laccase in phosphate buffer at pH = 7.1. The synthesis was performed in triplicate showing a clear reproducibility as it is shown in Figures S1 and S2. The reaction led to the formation of a brown-black pigment which was collected and extensively dried under a nitrogen flux. The powder was then used for all the experiments reported.

### 2.1. UV–Vis and FT-IR Characterization

**Figure 1:**
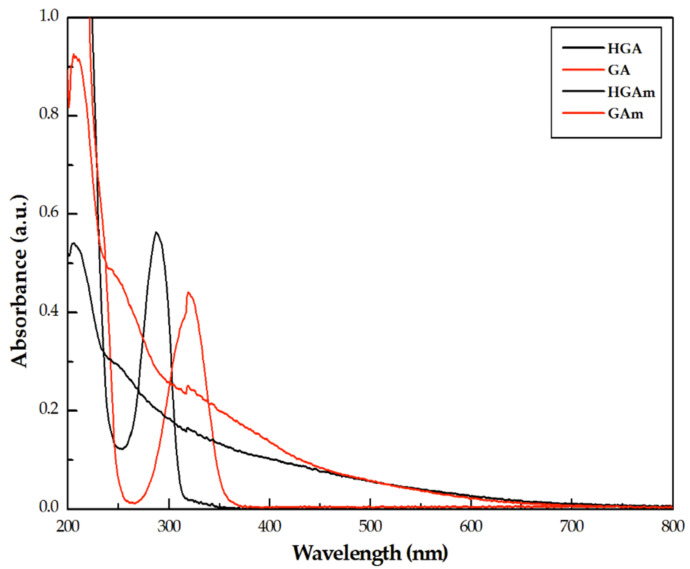
UV–vis spectra of HGAm (black line) and GAm (red line) paired with the corresponding UV–vis spectra of the monomers (HGA, black and GA, red). The measurements were carried out at room temperature (T = 298 K).

In Figure 1, the UV–vis spectra of the two samples of HGAm (black line) and GAm (red line) are reported and compared with the corresponding spectra of the monomers. The absence of the absorption peaks (at 295 nm and 320 nm for HGA and GA respectively), in the HGAm and GAm spectra, highlight that the oxidative enzymatic reaction was completed with the product formation. The UV–vis spectra of both pyomelanin-mimics are similar showing a broadband absorption typical of eumelanin pigments [21].

**Figure 2:**
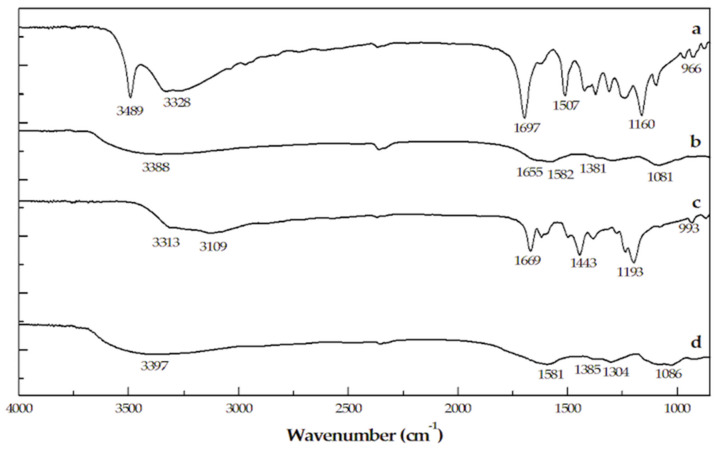
FT-IR spectra of (**a**) HGA monomer and (**b**) HGAm pigment, (**c**) GA monomer, and (**d**) GAm pigment.

In Figure 2 the IR spectra of the two pyomelanin mimics are compared with the ones of the monomers. At a first analysis, the FT-IR spectra of the polymers are less resolved showing that all the typical, well-defined peaks associated with HGA and GA are not present. In the case of the polymers, the spectral region 3500–3000 cm^−1^ corresponding to the -OH stretching band is smoothed out and shifted to higher frequencies [22]. The absence of this peaks in the spectra of the polymers is indicative that these groups are involved in new chemical bonds in the polymer structure. The spectra of the pyomelanins exhibit a shoulder at 1081–1086 cm^−1^ attributed to C-H in plane/out of plane bending mode. The broad peaks at 1381–1385 cm^−1^ and the peaks at 1582–1581 cm^−1^ are assigned to phenolic OH and aromatic C=C bending modes, respectively. The peak at wavenumber around 1650–1690 cm^−1^ (more evident in HGAm spectrum), corresponds to the C=O bond of the carboxylic acid functional group. In addition, the peaks at 966–993 cm^−1^ in HGA and GA, are absent in the polymers’ spectra. Peaks in this region correspond to aromatic C-H bonds and their absence in the melanin spectra suggests that in the pigments, aromatic units are linked to each other via C-C or C-O bonds [22,23].

### 2.2. Dynamic Light Scattering (DLS) and Nuclear Magnetic Resonance (NMR) Analysis

**Figure 3:**
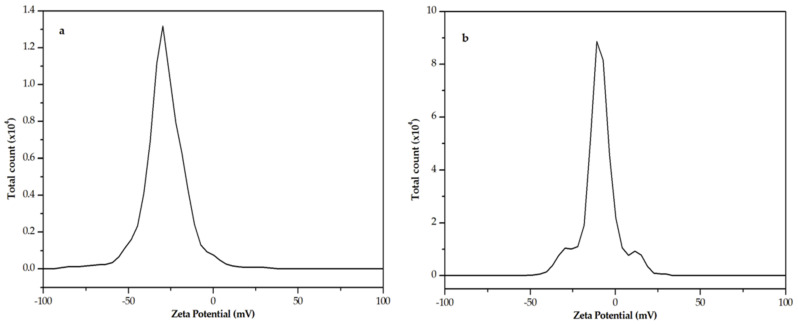
Zeta Potential of HGAm (**a**) and GAm (**b**) in water.

The structural characterization of melanins is a challenging task due to the heterogeneity and size of the samples [24,25]. DLS experiments (Figure S3) have been performed to determine the size of the polymers. Values of 13.1 kDa for HGAm and 11.5 kDa for GAm were determined. These gross values are in fair agreement with the data reported in literature for pyomelanin (10–14 kDa) [3]. The zeta potential values of −27.4 mV for HGAm and −8.5 mV for GAm, obtained in water, are reported in Figure 3.

^1^H NMR spectra of HGAm and GAm confirmed the disappearance of the corresponding monomers (Figure S4) [26]. In accordance with previous data reported for the gallic acid polymer, the aromatic part of the spectra was deprived of any resonance signals [27].

**Figure 4:**
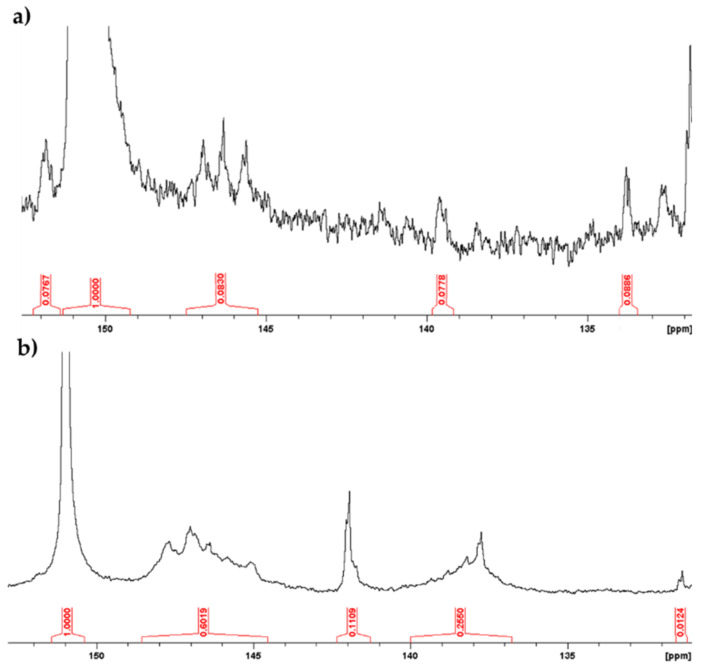
^31^P NMR of pyomelanin mimics. Spectra were recorded after derivatization of the samples as reported in reference [28]. (**a**) ^31^P NMR of HGAm. (**b**) ^31^P NMR of GAm.

^31^P NMR spectra were recorded by standard procedure [28] to evaluate the different type of OH subunits in the polymers. Data are reported in Figure 4 and Table 1.

HGAm and GAm showed appreciable amount of para-hydroxy phenyl units (136–140 ppm) besides to condensed units (151–152 ppm and 141–142 ppm) derived from the formation of the novel C-O and C-C linkages (Table 1). In addition, signals corresponding to residual COOH (134–136 ppm) and aliphatic OH groups (145–148 ppm) were also detected. The aliphatic OH signals may be due to a laccase mediated oxidation of the reactive benzylic positions, as well as, the occurrence of disproportionation of the COOH moiety [29].

The amount of condensed units was higher in GAm than HGAm, while a reversed trend was observed for the COOH groups (Table 1).

### 2.3. CW X-Band and Q-Band Pulse EPR Measurements

The HGAm and GAm powder samples were characterized by CW- X-band EPR at room temperature.

**Figure 5:**
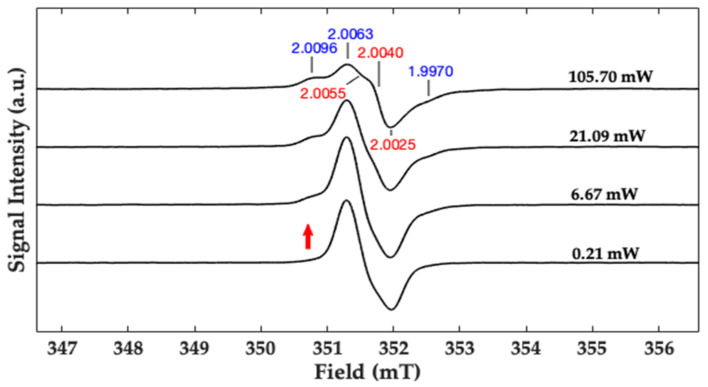
Room temperature (T = 298 K) X-band (ν = 9.86 GHz) EPR spectra of HGAm at pH 7.1 with a laccase:substrate molar ratio 1:1000 recorded at variable microwave power values.

In Figure 5, the EPR spectra of HGAm recorded at increasing microwave powers (from bottom to top) are reported. The signal recorded at lower microwave power (0.21 mW) is narrow without resolved hyperfine couplings with a g factor ~2.003. The X-band measurements show that, increasing the microwave power, a progressive saturation of the main signal with broadening and reduction in intensity is evident. Furthermore, when the microwave power is progressively increased, new features appear in the spectrum. The appearance and increasing intensity of a shoulder (indicated with the red arrow) paired with another evident signal at high field, are clearly evident at a microwave power of 105.70 mW. These new signals are indicative of the presence of at least two diverse radical species characterized by different relaxation times. The g-values matrix for the two species are shown in Figure 3. Considering the g values components, the two different radicals could be attributed to the presence of carbon-centred (g_xx_ = 2.0055, g_yy_ = 2.0040, g_zz_ = 2.0025, determined from Q-band measurements) and oxygen-centered radical species (g_xx_ = 2.0096, g_yy_ = 2.0063, g_zz_ = 1.9970). The g matrix values for the oxygen centred radical are better separated in the spectrum. This is due to the much larger anisotropy observed for phenoxyl radical (blue values), with large spin density on the oxygen and a large spin-orbit coupling, compared with radicals with spin density on carbon atoms [30,31]. The X-band EPR spectra, obtained in the same experimental conditions for GAm are reported in Figure S5.

In this context, the use of a multifrequency EPR approach has been used to gain more information on the nature of the different radical species [32]. At higher frequency various contributions with different g values may be better separated and assigned. The Q-band pulse measurements are used for the characterization of the longitudinal relaxation times of the radical signal in each sample.

**Figure 6:**
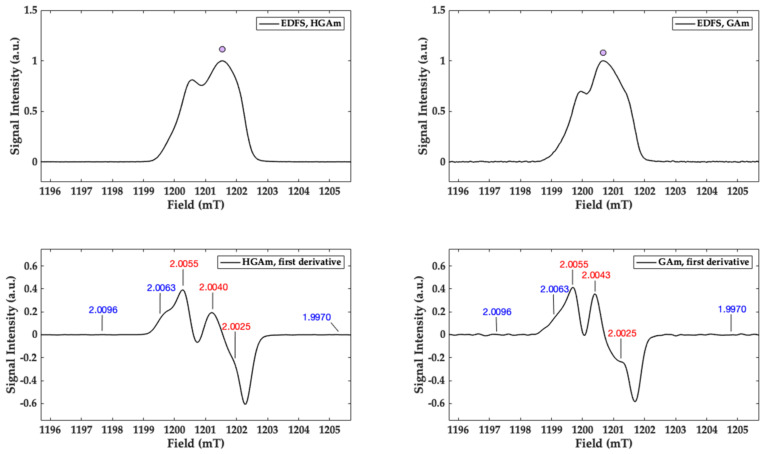
Q-band (ν = 33.67 GHz) EPR field swept echo detected spectrum (EDFS) recorded at 298 K for the HGAm sample (left panel, up) and the corresponding pseudo field modulation spectrum calculated (left panel down) are reported and compared with the corresponding ones obtained for the GAm pigment (right panel) (ν = 33.67 GHz). Rectangular pulses were used for the Hahn echo sequence π-τ-π/2, with pulse lengths π = 76 ns and π/2 = 38 ns. The g matrix values for the radical species are reported.

In Figure 6 the Q-band (ν = 33.67 GHz) EPR field swept echo detected spectrum (EDFS) of the HGAm sample (left panel, up) and the corresponding derived pseudo field modulation spectrum calculated (left panel, down) are reported and compared with those obtained for the GAm pigment (right panel). In these spectra, the g values matrix, for the carbon-centred radical, are resolved and shown. This combined EPR approach has clearly shown that for HGAm and GAm, the carbon centred radicals are in presence with oxygen radical species as semiquinone fraction, differently from what it is commonly reported in literature where only the quinoinoid structure is reported for the HGAm pigment [3].

**Figure 7:**
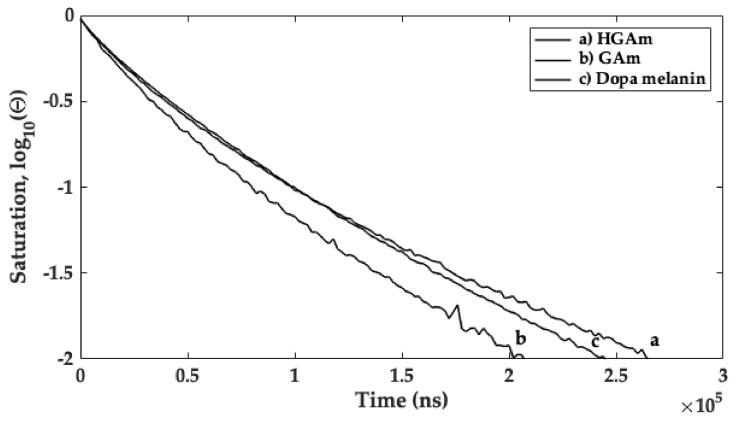
Q-band saturation curves, Θ/Θ_0_ (Θ_0_ = 1), obtained from the PFSR experiment performed at room temperature (298 K) on the (**a**) HGAm, (**b**) GAm, and (**c**) Dopa melanin samples, for the determination of T_1_ relaxation times.

The HGAm, GAm longitudinal relaxation times, and T_1_, were then studied using Q-band picket fence saturation recovery (PFSR) experiments at room temperature (298 K). This experiment was performed to assess the relaxation times, as discriminant physical property for melanin and melanin-like pigment characterization [33]. The PFSR technique was used instead of other techniques, in order to minimize the effect of spectral diffusion in relaxation times determination. The field region corresponding to the maximum of absorption in the EDFS spectra (Figure 6, pink circle) was selected to run the PFSR experiment. The saturation recovery curves, and the obtained longitudinal relaxation times, are reported in Figure 7 and Table 2, together with the results obtained for the Dopa melanin sample used as comparison. A biexponential model was employed to address the presence of other effects, as spectral diffusion, contributing to the longitudinal relaxation (weight factor A_1f_ and time constant T_1f_), occurring together with the spin-lattice relaxation process (represented by the weight A_1s_ and the time constant T_1s_).

As the results of a biexponential decay model for data analysis, the T_1_ values obtained from the most intense peak (mainly assigned to the relaxation time for the C centred radical species) in the EDFS spectra of the GAm and HGAm are coherent with the T_1_ values obtained for dopa-melanin sample previously studied using the same experimental contour [33].

### 2.4. Antioxidant Properties and Scanning Electron Microscopy Analysis

Natural melanins and related synthetic polymers show high antioxidant activity. A correlation between the paramagnetic properties and antioxidant activity of poly-phenolic polymers has been demonstrated [19,34]. In Table 3 the EC_50_ value has been estimated for both the pyomelanin mimic samples and compared with the EC_50_ value for the gallic acid polymer used as reference. The EC_50_ has been determined by the DPPH assay using the EPR and the UV–vis techniques. The DPPH assay is commonly used to measure the efficiency of electron transfer process. HGAm sample shows the lower value of the EC_50_ demonstrating the higher antioxidant activity compared to GAm and the gallic acid polymer. The high antioxidant activity for HGAm hampered to determine its value by UV–vis technique as the absorbance value was not determined with precision due to the overlapping of the absorption curves. The antioxidant activity determined for HGAm is also higher compared to that determined for other melanin and melanin-mimic pigments [11].

**Figure 8:**
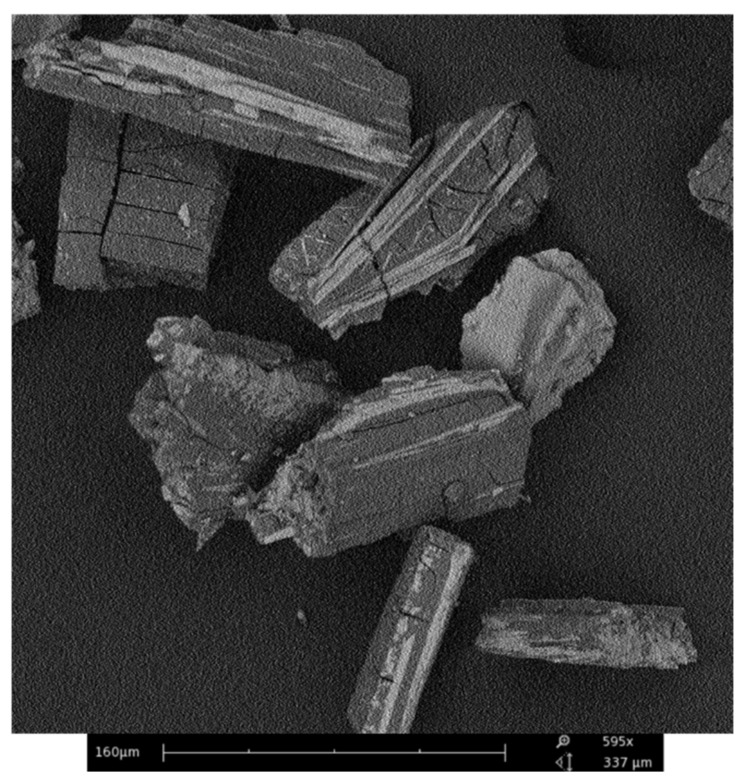
SEM image of HGAm sample.

At the end the morphological analysis was performed. In Figure 8 the SEM image of the HGAm powder is shown. The nanoaggregates show different overlapping planar sheets pointing out to an extended π–π aromatic interaction system [35].

## 3. Discussion

In this paper, the enzymatic synthesis of HGAm and GAm at physiological pH has been performed. New water soluble pyomelanin-mimics with high antioxidant properties have been obtained. These mimics show physico-chemical characteristics similar to the insoluble eumelanin-like materials. Recently a homogentisic acid derived pigment has been proposed as a biocompatible label for optoacoustic imaging of macrophages and it is used as a reference to obtain more insights on composition and collagen disruption in alkaptonuria cartilage [5,12]. Laccases are oxidative enzymes able to oxidize a wide variety of molecules comprising polyphenols, aromatic amines etc. [36]. Hydroquinones are very reactive molecules and their enzymatic oxidation prompts to the formation of the semiquinone radical which represents the reactive species towards the formation of the fully oxidized quinone and reduced quinol species (see Scheme S1). The following polymerization reaction is enzyme independent. Phenolic compounds, monomers and polymers, are going to be studied for their antioxidant properties and applications in different fields ranging from food to biomaterial functionalization. The antioxidant activities is mainly due to the capacity of this class of compounds to donate electrons or protons with the consequent extensive electron delocalization with the formation of π-conjugation and supramolecular structures [19]. The persistent paramagnetism of this structure enables the use of the EPR as the election technique for their characterization [18]. The eumelanin EPR signal is commonly attributed to the concomitant presence of a carbon centred (g~2.0032) and semiquinone (g~2.0045) radical species contributing to the EPR lineshape depending on hydration level and pH of the sample [17]. The UV–vis analysis shows that the HGAm and GAm pigments have a spectrum profile with absorption in the visible region and high intense absorption in the UV region. The CW X-band EPR spectra show a clear presence of two different radical species at increasing microwave power and the g-values matrix representing the two species are indicated in red (carbon-centred radical) and in blue (oxygen-centred radical species) in Figure 5 and Figure 6. Oxygen radical species are characterized by a greater anisotropy due to the large spin orbit coupling of the oxygen atom compared to carbon or nitrogen. This is the reason why also at X-band the g-matrix values are separated and readable in Figure 5 (blue values). The carbon g-matrix values are indicated in Figure 5 but they are overlapped in the central region of the EPR spectrum (red values) at X-band. The use of higher frequencies in the so-called EPR multifrequency approach, allows to separate and obtain the g-values for the carbon-centred radical species. Here we have performed pulse Q-band experiments as reported in Figure 6 where the EDFS spectra paired with the corresponding pseudo field modulation spectra calculated for both the samples are reported. In Figure 6 the g_xx_ and g_zz_ values for the oxygen-centred radical are indicated but not visible in the calculated spectrum. The PFSR experiments were also performed on both samples and the T_1_ relaxation time, represented by T_1s_, determined and compared with the one recorded previously for the eumelanin sample [33]. The T_1_ relaxation time values for our samples are in agreement with the one determined recently for polydopamine radicals [37], even if the differences in sample preparations have to be taken into account. The T_1_ value of the HGAm was found to be higher than that of dopa-melanin, and different from that reported for the GAm, which was in turn lower than it. Overall, the value of T_1s_ of HGAm was found to be about 49% greater than that of GAm. It is interesting to point out that, in our case, the higher antioxidant activity of HGAm is paired with a longer longitudinal relaxation time, while the faster relaxation determined for the GAm sample is paired with a higher EC_50_ value and lower antioxidant activity. This evidence will be studied with further experiments to assess a possible relationship between the two properties in HGAm and GAm.

Furthermore, the presence of p-hydroxyphenyl units as determined by NMR measurements, support the presence of oxygen radical centers in both samples.

These results, taken together, show that the HGAm and GAm pigments have a common radical composition where a carbon-centered radical species is paired with the presence of an oxygen based radical species more probably due to semiquinone moieties in lower percentage. The quinoinoid structures are involved in a π-conjugate supramolecular assembly where the electrons can move not only along the polymer chain but also interchains [35,38]. This analysis shows also that CW Multifrequency EPR with pulse EPR relaxation times measurements could be employed as investigation tool to expand the current knowledge on radicals structural and dynamical properties in melanin and melanin-like pigments, where solely X-band EPR methodology is commonly employed for their characterization. Open questions regarding the actual structure of the radical species found in melanin and melanin-like pigments, and their mesoscale structural organization can be solved by this combined approach contributing to link the molecular-level structural framework of melanin pigments with their macroscopic physical and chemical properties.

## 4. Materials and Methods

### 4.1. Synthetic Procedure and Spectrophotometric Analysis

Pyomelanin mimics from HGA and GA were synthesized following the protocol reported in [21]. The synthesis was performed using *T. versicolor* laccase (0.1 mM–0.92 U mg^−1^) at pH 7.1 in phosphate buffer (100 mM) and with a laccase:substrate 1:1000 molar ratio for both of them. The reaction mixture was leave to react for 48 h in a shaker (150 rpm) at room temperature (T = 298 K) in presence of oxygen. A brownish solution was obtained. The powder was collected after centrifugation and extensively dried under a nitrogen flux. The synthesis was performed in triplicate. The substrates and solvents were obtained from Sigma–Aldrich (Milan, Italy) and used without further purification.

The UV–vis spectra were obtained using a Lambda 900/Perkin Elmer Instruments (Norwalk, CT, USA) spectrophotometer operating in the range 200–800 nm. ATR (Attenuated Total Reflectance) Fourier Transform Infrared spectra were obtained using a Perkin Elmer Spectrum One spectrometer. Spectra were acquired in the wavenumber range of 500–4000 cm^−1^ at room temperature using KBr with a resolution of 4 cm^−1^.

### 4.2. DLS and NMR Analysis

The dynamic light scattering experiments were performed with a Zetasizer NanoZS90 instrument (Malvern Instruments Ltd, Worcestershine, UK). HGAm and GAm were solubilised in distilled water with a concentration of 0.5 mg/mL. Three independent measurements were carried out and used in the analysis of the data.

^1^H NMR spectra were recorded after solubilization of the sample (11.0 mg) in D_2_O (1.0 mL) and the spectra were recorded on a Bruker 400 MHz spectrometer. ^31^P NMR experiments were recorded as reported in [28]. Briefly, the sample (20 mg) was solubilized in dimethylformamide/pyridine/CDCl_3_ mixture (0.8 mL; 1.0:1.5:2.5 ratio) and treated with 2-chloro-4,4,5,5-tetramethyl-1,3,2-dioxaphospholane (TMDP) (50 μL) in the presence of n-hydroxy-5-norbornene-2-3-dicarboximide (NHND) (0.9 mmol) as internal standard and chromium(III) acetylacetonate (3.0 mg) as relaxing agent. The spectra were recorded with a Bruker 400 MHz spectrometer overnight.

### 4.3. Multifrequency Continuous Wave (CW) and Pulse EPR Analysis

EPR spectra were measured with a Bruker ELEXSYS E580E Super Q-FT spectrometer, for CW (X- and Q-band) and pulse Q-band EPR measurements. CW X-band spectra were performed using a Bruker ER 049X microwave bridge with 4122SHQE/0208 cavity. All samples were investigated in powder form. The pyomelanin dry powders were inserted within cylindrical suprasil capillaries (WG-222T-RB, Cortecnet Europe, Les Ulis, France) with ID x OD equal to 1.1 × 1.6 mm and used for both X- and Q-band measurements. The X-band experimental conditions for EPR spectra acquisition were as follows: 9.8 GHz microwave frequency, 0.1 mT modulation amplitude and variable microwave power values in the range (0.0002–210.8 mW) for the power saturation measurements. For an accurate determination of g-factor, two standard markers were used (one with a g = 2.0028 and the other with g = 1.9800). The EDFS spectra of the melanin samples were acquired with a π/2-τ-π echo sequence. A PFSR sequence was employed for all the measurement of longitudinal relaxation times.

### 4.4. EC_50_ Determination

The antioxidant activity was determined with the EC_50_ assay carried out with EPR and UV–vis spectroscopies. For the EPR experiments, a stock solution of DPPH 0.4 mM in ethanol with a fixed volume of 100 μL for each sample was used. The antioxidant was dissolved in phosphate buffer with a concentration of 0.127 mg/mL for gallic acid, 1.77 mg/mL for GAm, 0.26 mg/mL for HGAm and 0.59 mg/mL for the gallic acid polymer. For each of these substances an increasing volume of solution ranging from 5 μL to 100 μL was added to DPPH solution to reach a final volume of 200 μL. The DPPH radical signal was monitored for all samples and the DPPH radical in the absence of antioxidant is the reference signal for the scavenger radical percentage determination. After the spectra acquisition, the double integral of each spectrum has been calculated and the scavenger effect percentage was determined using the following formula

$$\mathrm{Scavenger}\;\mathrm{effect}\;\%\;=\;\frac{A_{0}-A_{a}}{A_{0}}\cdot 100$$

where A_0_ represents the double integral of the DPPH radical without the addition of the antioxidant, A_a_ is the double integral of the DPPH radical after the addition of antioxidant. Spectra acquisition was run at a fixed time of 15 min after the addition of the antioxidant solution to the DPPH sample.

The antioxidant activity, for the UV–vis DPPH test, was evaluated monitoring the reduction of the DPPH radical peak at 520 nm, for a fixed reaction time of 15 min at T = 298 K in presence of different concentrations of antioxidants. The samples were prepared with 200 μL of DPPH (0.2 mM–79 μg/mL) and 200 μL of antioxidant at variable concentration. The pyomelanin mimics powder were dissolved in phosphate buffer with a concentration of 0.1 mg/mL for HGAm and 0.16 mg/mL for GAm and gallic acid polymer. For each of these antioxidants an increasing volume of solution ranging from 20 μL to 200 μL was added to the DPPH solution to reach a final volume of 400 μL.

Plotting the DPPH scavenger percentage in function of the log of antioxidant concentration expressed in μg/mL, the EC_50_ value was automatically calculated using GraphPad Prism 5.01. Log (inhibitor) vs. normalized response (variable slope) was the statistical model used for data elaboration of DPPH^.^ assay [39]. The obtained results, for the EPR analysis, are reported in supporting information (Figures S6–S8). All measurements were repeated in triplicate and the standard deviation error for each sample was calculated.

### 4.5. Scanning Electron Microscopy (SEM) Analysis

The morphological analysis was performed using the Scanning Electron Microscope (SEM; Phenom G2 pure desktop apparatus) (Thermo Fisher Scientific, Waltham, MA, USA) working in the magnification range 20–17,000×.

## Figures and Tables

**Table 1:** Functional groups distribution (mmol/g) in HGAm and GAm polymers ^a^.

Sample	Carboxylic Acid	Aliphatic-OH	CondensedPhenolic Units	P-hydroxyphenyl Units
HGAm	4.10	3.98	1.6	1.9
GAm	0.52	31.47	5.64	13.64

^a^ Quantitative ^31^P NMR spectra of HGAm and GAm. The samples were recorded after derivatization with 2-chloro-4,4,5,5-tetramethyl-1,3,2-dioxaphospholane (TMDP) using n-hydroxy-5-norbornene-2 3-dicarboximide (NHND) as internal standard in DMF/Pyridine.

**Table 2:** Longitudinal relaxation times obtained for HGAm, GAm, and Dopa melanin samples, recorded at room temperature. A biexponential decay was used to interpret the saturation recovery results: $y=A_{f}\exp\left(-\frac{t}{T_{1f}}\right)+{\;A}_{s}\exp\left(-\frac{t}{T_{1s}}\right)+c$. An experimental error of ±3 µs can be considered in the data reported for the HGAm and Dopa melanin data, while an experimental error of ±5 µs has been considered for GAm.

	T_1s_ (μs)	T_1f_ (μs)	A_f_/A_s_
HGAm	254	59	1.51
GAm	170	44	1.13
Dopa melanin	216	61	1.50

**Table 3:** EC_50_ (μg/mL) values from DPPH assay.

Sample	EPR	UV–vis
HGAm	2.7 ± 0.7	n.d.
GAm	27.2 ± 5.4	25.7 ± 2.6
Gallic acid polymer	14.4 ± 3.3	12.7 ± 4.7

## Data Availability

Data is contained within the article and supplementary material.

## Supplementary Materials

The following are available online at https://www.mdpi.com/1422-0067/22/4/1739/s1.
