# Supplementary material for: Homogentisic Acid and Gentisic Acid Biosynthesized Pyomelanin Mimics: Structural Characterization and Antioxidant Activity
Source: Int J Mol Sci. 2021 Feb 9;22(4):1739. doi: 10.3390/ijms22041739 (PMC7916096; doi:10.3390/ijms22041739)
Supplement: Supplementary file 1 [file ijms-22-01739-s001.pdf]

Supplementary material

Homogentisic acid and gentisic acid biosynthesized pyomelanin mimics: structural characterization and antioxidant activity

Maher Al-Khatib [1], Jessica Costa, [1] Daniele Spinelli, Eliana Capecchi[3], Raffaele Saladino[3], Maria Camilla Baratto[1] and Rebecca Pogni [1,*]

[1] Department of Biotechnology, Chemistry and Pharmacy, Via A. Moro 2, 53100 Siena; maher.al@unisi.it, jessica.costa2@unisi.it , mariacamilla.baratto@unisi.it, rebecca.pogni@unisi.it
[2] Next Technology Tecnotessile, Via del Gelso, Prato (Italy) 2; chemtech@tecnotex.it
[3] Department of Ecology and Biology, University of Tuscia, 01100 Viterbo, Italy; e.capecchi@unitus.it, saladino@unitus.it

* Correspondence: rebecca.pogni@unisi.it;

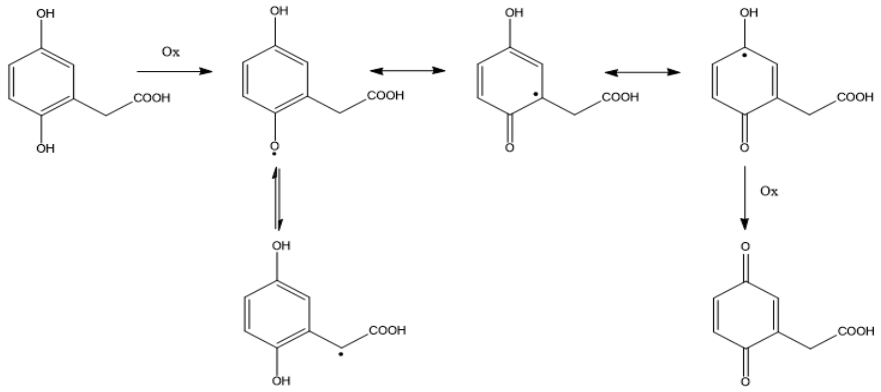

Scheme 1 – Scheme of the radical delocalization in HGA

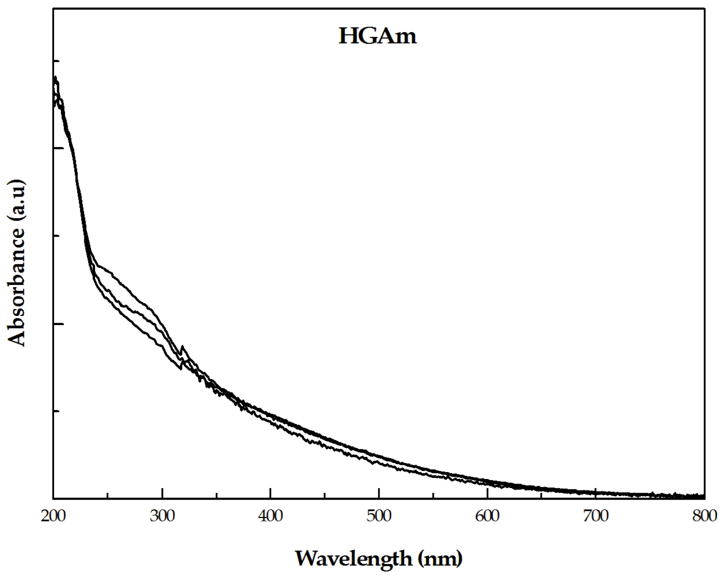

Fig S1: Reproducibility test of pyomelanin mimic from homogentisic acid (HGAm). The synthetic conditions were the same for all samples (pH 7.1 and molar ratio lac:HGA 1:1000). The UV-vis measurements were carried out at room temperature.

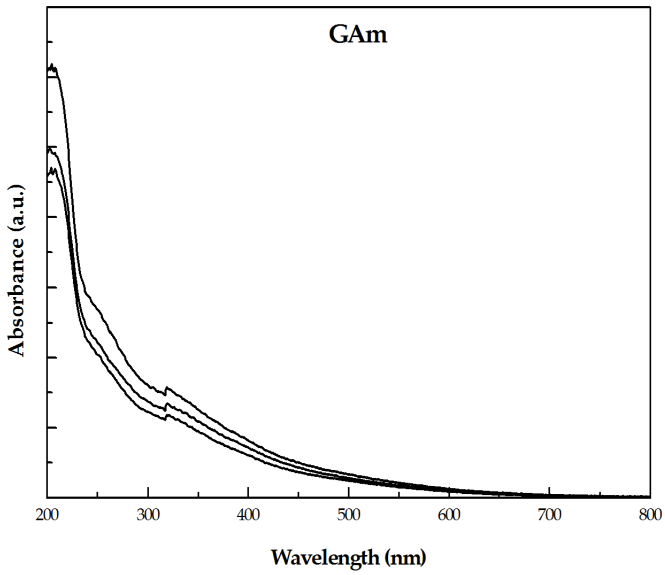

Fig S2: Reproducibility test of pyomelanin mimic from gentisic acid (GAm). The synthetic conditions were the same for all samples (pH 7.1 and molar ratio lac:GA 1:1000). The UV-vis measurements were carried out at room temperature.

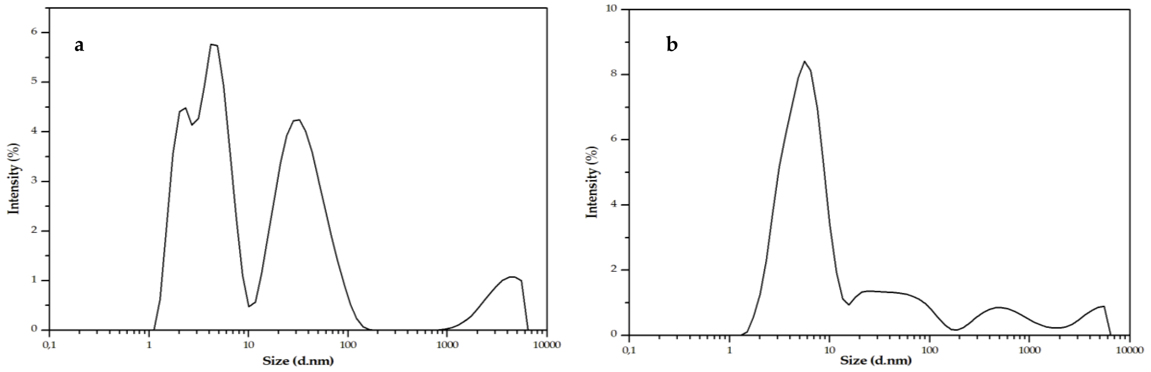

Fig. S3: DLS of a)HGAm and b) GAm. The spectra have been recorded in water.

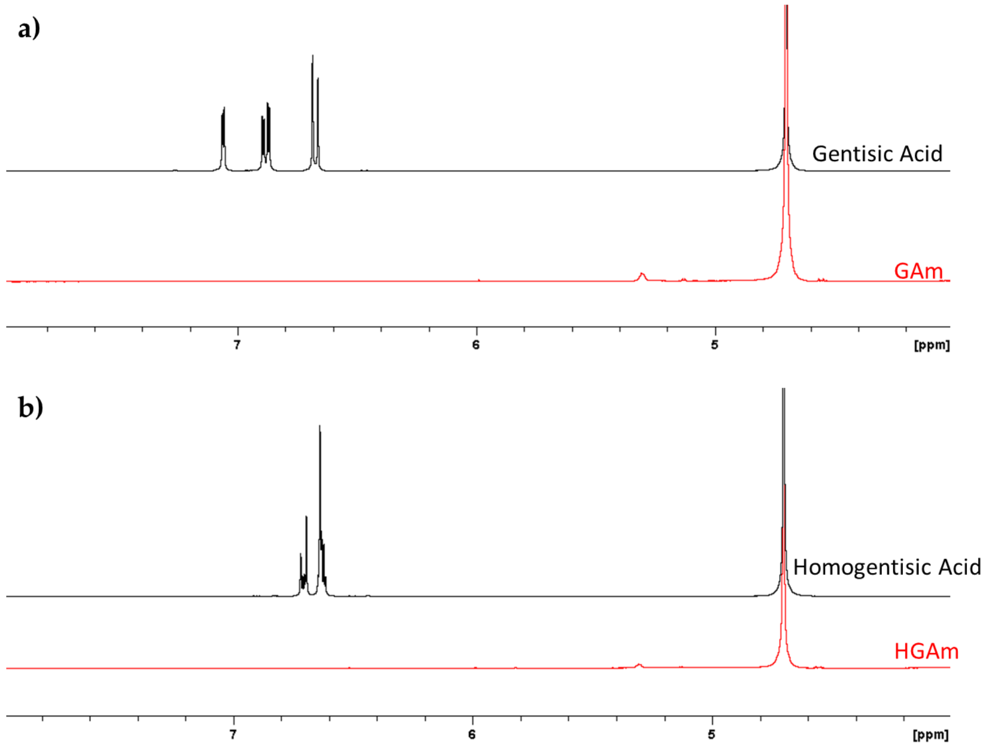

Fig. S4: In panel A: ^{1}H NMR spectra of GA (black line) and GAm (red line). In panel B: ^{1}H NMR spectra of HGA (black line) and HGAm (red line).

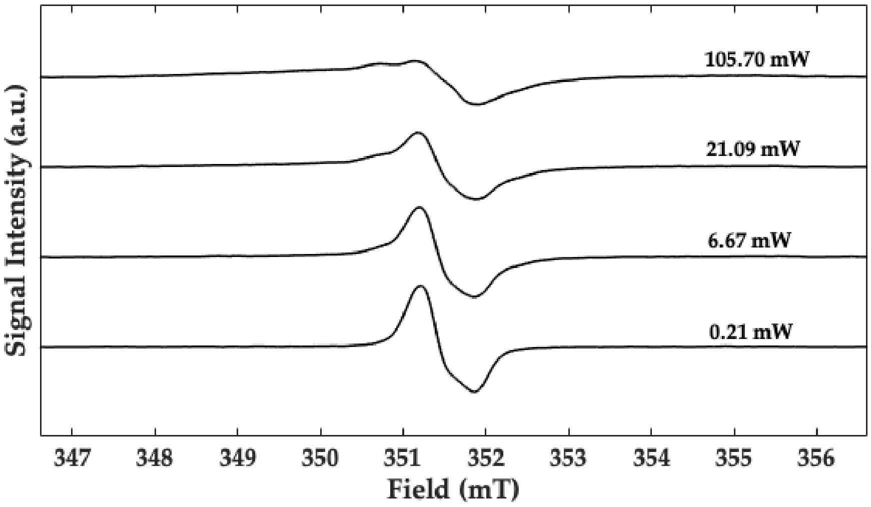

Fig. S5: Room temperature X-band (ν= 9.86 GHz) EPR spectra of GAm at pH 7.1 recorded at variable microwave power values.

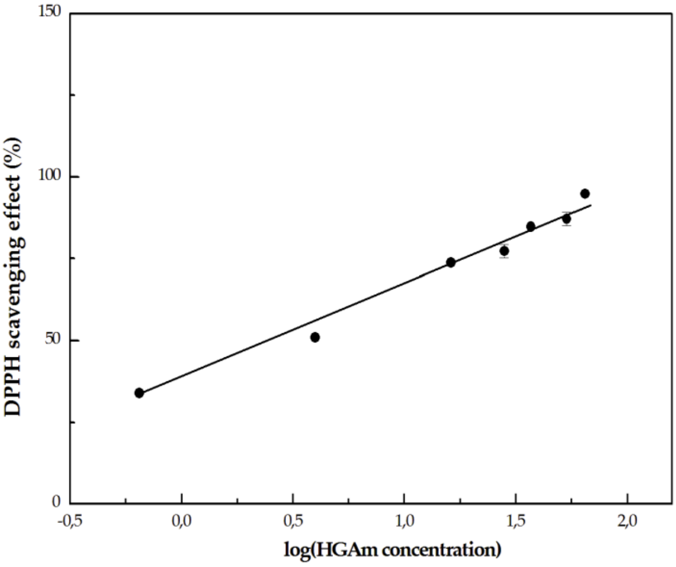

Fig. S6 Antioxidant activity of HGAm. The EC_{50} value was calculated using GraphPad Prism plotting the DPPH scavenger percentage measured by EPR spectroscopy in function of the log of HGAm concentrations analyzed. All measurements were repeated in triplicate.

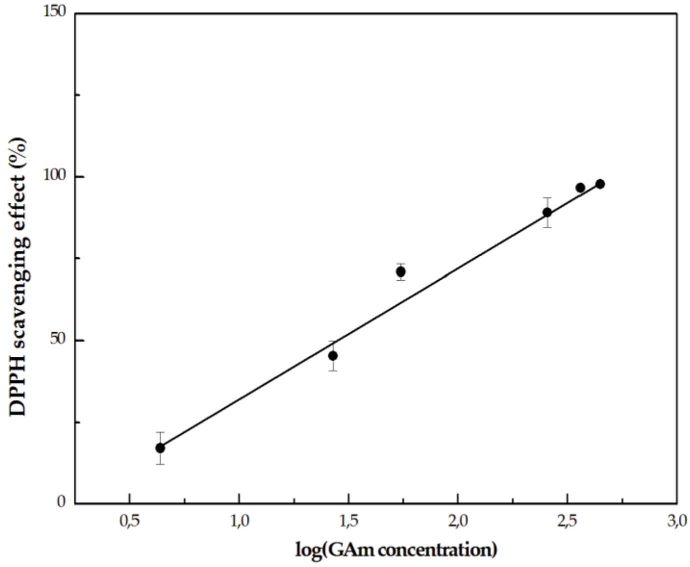

Fig. S7 Antioxidant activity of GAm. EC_{50} value was calculated using GraphPad Prism plotting the DPPH scavenger percentage measured by EPR spectroscopy in function of the log of GAm concentrations analyzed. All measurements were repeated in triplicate.

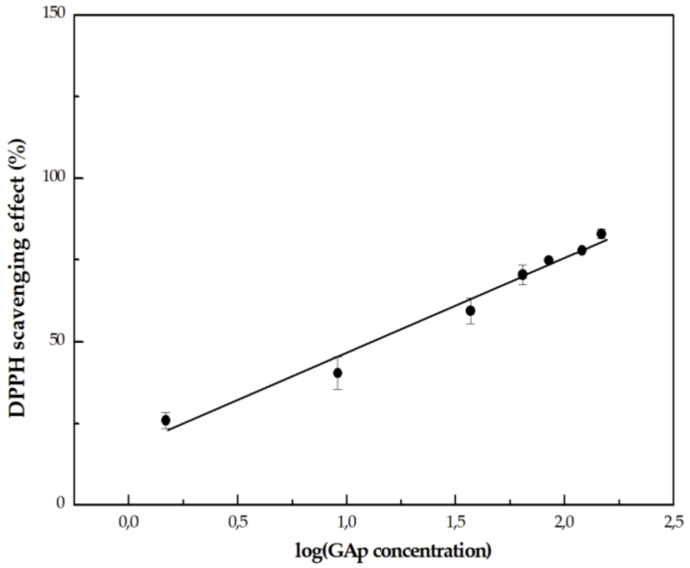

Fig. S8 Antioxidant activity of gallic acid polymer (GAp). The EC_{50} value was calculated using GraphPad Prism plotting the DPPH scavenger percentage measured by EPR spectroscopy in function of the log of GAp concentrations analyzed. All measurements were repeated in triplicate.
